# *Uncaria tomentosa*—Adjuvant Treatment for Breast Cancer: Clinical Trial

**DOI:** 10.1155/2012/676984

**Published:** 2012-06-28

**Authors:** Maria do Carmo Santos Araújo, Iria Luiza Farias, Jessie Gutierres, Sergio L. Dalmora, Nélia Flores, Julia Farias, Ivana de Cruz, Juarez Chiesa, Vera Maria Morsch, Maria Rosa Chitolina Schetinger

**Affiliations:** ^1^Department of Chemistry, Federal University of Santa Maria, Avenida Roraima, Predio18, 97105-900 Santa Maria, Rs, Brazil; ^2^Santa Maria University Hospital, Federal University of Santa Maria, Avenida Roraima, Predio18, 97105-900 Santa Maria, Rs, Brazil; ^3^Department of Biology, Federal University of Santa Maria, Avenida Roraima, Predio18, 97105-900 Santa Maria, Rs, Brazil; ^4^Department of Industrial Pharmacy, Federal University of Santa Maria, Avenida Roraima, Predio18, 97105-900 Santa Maria, Rs, Brazil; ^5^Department of Morphology, Federal University of Santa Maria, Avenida Roraima, Predio18, 97105-900 Santa Maria, Rs, Brazil

## Abstract

Breast cancer is the most frequent neoplasm affecting women worldwide. Some of the recommended treatments involve chemotherapy whose toxic effects include leukopenia and neutropenia. This study assessed the effectiveness of *Uncaria tomentosa* (Ut) in reducing the adverse effects of chemotherapy through a randomized clinical trial. Patients with Invasive Ductal Carcinoma—Stage II, who underwent a treatment regimen known as FAC (Fluorouracil, Doxorubicin, Cyclophosphamide), were divided into two groups: the UtCa received chemotherapy plus 300 mg dry Ut extract per day and the Ca group that only received chemotherapy and served as the control experiment. Blood samples were collected before each one of the six chemotherapy cycles and blood counts, immunological parameters, antioxidant enzymes, and oxidative stress were analyzed. *Uncaria tomentosa* reduced the neutropenia caused by chemotherapy and was also able to restore cellular DNA damage. We concluded that Ut is an effective adjuvant treatment for breast cancer.

## 1. Introduction

Breast cancer is the most frequent neoplasm affecting women worldwide, both in terms of incidence and mortality. The disease is more common in developed countries with its highest incidence being observed in the United Kingdom, Australia, USA, and Canada. From invasive tumors, ductal carcinoma and its variants represent 80% of cases [1] and the proportion of women with tumors in clinical stages I and II increased from 41 to 65% in the last decade [1, 2]. About 70% of breast cancers express Estrogen hormone receptors and/or Progesterone receptor [3]. These markers along with the HER-2 receptor (c-erbB2) provide information about the tumor and how it might respond to different treatments [4].

 Chemotherapy is among the recommended treatments for breast cancer, which can be a single or combination therapy with multiple drugs. Chemotherapy drugs have very narrow therapeutic indexes in terms of nonselective toxic effects on normal tissues, with neutropenia being the most frequently observed adverse reaction, which increases the risk of infections [5].

Pharmacological interventions that reduce or prevent adverse effects may have a substantial impact on cancer treatment. According to World Health Organization (WHO), 80% of the population use medicinal plants as alternative or complementary procedures for the treatment of their diseases [6].

Studies have reported the use of herbal medicines in cancer patients to minimize the effects of chemotherapy. *Uncaria tomentosa *(Utor Cat's Claw) is a medicinal herb that has been used in the treatment of different diseases including cancer. Patients who use Cat's Claw along with traditional cancer therapies, such as chemotherapy and radiation, reported fewer adverse effects to those therapies [7]. Uthelps in the restoration of cellular DNA, preventing mutations and cell damages caused by chemotherapy drugs [8]. It modulates the activity in the immune system, such as the proliferation of normal T and B lymphocytes [9], also modulating certain cytokines, including IL-1 and IL-6, TNF-*α* [10]. In addition, it has antioxidant properties [11]. Its direct myelostimulating effects, through myelopoiesis stimulation and Colony-Stimulating Factors (G-CSF) [8, 12], seem to be a beneficial option to minimize the risks associated with neutropenia.

Numerous reports present a theoretical understanding of Ut action mechanisms, but none of these studies consisted of clinical trials. Thus the objectives of this study are situated in this context, which consisted of a clinical trial using *Uncaria tomentosa* Herbarium tablets, as adjuvant treatment for breast cancer.

## 2. Methods

### 2.1. Design and Patients

A randomized interventional study was performed. It was carried out with 40 patients who had undergone complete breast cancer resection, which was histologically diagnosed as Invasive Ductal Carcinoma—Stage II [2], and who were going to begin adjuvant chemotherapy with Doxorubicin-based scheme for six cycles, at the Santa Maria University Hospital, Brazil.

 Patients were randomly divided into two groups: the CaUt group, which was treated with six cycles chemotherapy + Ut and the cancer group (Ca), which only received six cycles of chemotherapy, according to the date treatment was started, as follows: the first patient who agreed to participate in the study was included into the CaUt group, the second, into the Ca group, and, thus, successively, until the end.

For the control group were invited to participate healthy women, classified by clinical trial, with similar age of the patients and that did not receive any medication in the last 30 days or have chronic disease.

Patients were part of the study during 6 chemotherapy cycles, of 21 days each. Medication dosage in the CaUt group was as follows: FAC (Fluorouracil, Doxorubicin, and Cyclophosphamide) and 3 tablets of Ut (Unha de Gato Herbarium), daily, from day 2 to day 21. The dose of Ut was similar to that used in previous studies, with 250–350 mg C-MED-100, in aqueous Ut extracts [13].

The calculation to estimate the sample size required for randomized clinical trial was performed according to Greenberg et al. [14], with constant significance level (*α*) of 5%, and statistical power of 90% (*β* 10%), using as reference the studies of Sheng et al. [15].

The Human Ethics Committee of the Santa Maria University Hospital, Brazil, approved the present study and informed consent was obtained from all participants (protocol number: 0169.0.0242.000-07.). All subjects were invited to participate and were informed in detail about the design of this study through a Statement of Consent signed by the researcher and participants. They were informed that they could be selected randomly for the Ca or UtCa group.

### 2.2. Materials

Each tablet of *Unha de Gato* Herbarium contained 100 mg of dry *Uncaria tomentosa* extract. Biological materials used in the tablets were derived from plants in their natural habitat. The *Uncaria tomentosa* extract was prepared by Ultra-turrax Extraction (Biotron, Kinematica AG) from ground bark (Centroflora) using 70% ethanol (Dipalcool). The HPLC analysis of the Ut dry extract presents 2.57% pentacyclic oxindole alkaloids (POAs) content, which was calculated with reference to external calibration curves of mitraphylline. The extract analysis showed absence of tetracyclic oxindole alkaloids in the sample, allowing its use for therapeutic and research purposes in accordance with the U.S. Pharmacopeia.

### 2.3. Sample Collection

Blood was collected into citrate, EDTA, heparin Vacutainer tubes, without any anticoagulants, before chemotherapy and after each of the 6 cycles.

### 2.4. Biochemical Parameters

A COBAS INTEGRA system was used for the quantitative determination of the blood chemical constituents, and data were acquired through a COBAS INTEGRA 400 Plus apparatus (USA).

### 2.5. Hemograms

Blood samples were analyzed using a Pentra apparatus (France). The lowest values were confirmed by observation of slides, using a May Grünwald-Giemsa Stain and optical microscopy.

### 2.6. CD3+, CD4+, and CD8+ Cells

Samples were collected in EDTA and analyses were performed using a three-color fluorescence-activated cell sorter (FACSCalibur, Becton Dickinson Biosciences, United States) and a Multiset software (Becton Dickinson). FITC-conjugated anti-CD4, PE-conjugated anti-CD8, and PerCP-conjugated anti-CD3 were used. Immune subpopulations were measured as a percentage of the total CD3+ cell number.

### 2.7. Interleukin 6 (IL-6)

ELISA assays of IL-6 were carried out according to a previously published method [16], at room temperature in Microtiter 96-Well Plates (Nunc-Immuno Plate MaxiSorp) and optical densities (O.D.) at 490 nm, which were determined using a Microplate Reader (Thermo Scientific Multiskan FC, Vantaa, Finland).

### 2.8. Single Cell Gel Electrophoresis (Comet Assay)

The alkaline comet assay was performed as described by Singh et al. [17] in accordance with the general guidelines for use of the comet assay [18, 19]. Lymphocytes were suspended in 0.7% low-melting-point agarose and phosphate-buffered saline (PBS) at 37°C and placed on microscopic slides with a layer of 1% agarose. The slides were immersed in lysis solution at 4°C for 1 h and followed by electrophoresis at 25 V, 300 mA, for 40 min at steady temperature. The slides were then silver-stained, as described by Nadin et al. [20]. All steps, from sample collection to electrophoresis, were conducted under yellow light to minimize the possibility of cellular DNA damage. One hundred cells (50 cells from each of the two replicate slides) were selected and analyzed. Cells were visually scored according to tail length and received scores from 0 (no migration) to 4 (maximal migration). Therefore, the damage index for cells ranged from 0 (all cells with no migration representing a damage index of 0%) to 400 (all cells with maximal migration, representing a damage index of 100%). The slides were analyzed under blind conditions by at least two different individuals [21].

### 2.9. Carbonylation of Serum Protein

The carbonylation of serum proteins was determined by a modified Levine's method [22]. The absorbance of the supernatant at 370 nm was measured using a spectrophotometer. Carbonyl content was calculated using 22 × 10^3^ mM^−1^ cm^−1^ as the molar extinction coefficient, and the results were expressed as nanomoles of carbonyl groups per milligram protein.

### 2.10. Determination of Lipid Peroxidation

Lipid peroxidation was estimated by measuring TBARS levels in plasma samples according to a modified method of Jentzsch et al. [23]. The concentration of malondialdehyde (MDA) was determined by measuring the absorbance at 532 nm using a spectrophotometer. The results were expressed as nanomoles of MDA per milliliter of plasma.

### 2.11. Catalase (CAT) and Superoxide Dismutase (SOD) Activities

CAT activity was determined in accordance with a modified method of Nelson and Kiesow [24]. The change in absorbance at 240 nm was measured for 2 min. CAT activity was calculated using the molar extinction coefficient (0.046 mM^−1^ cm^−1^), and the results were expressed as picomoles of CAT per milligram of protein.

SOD activity was determined based on the inhibition of the radical superoxide reaction with adrenaline as described by McCord and Fridovich [25]. SOD activity is determined by measuring the rate of adrenochrome formation, observed at 480 nm, in a medium containing glycine-NaOH (50 mM, pH 10) and adrenaline (1 mM).

### 2.12. Statistics

Results are expressed as mean ± standard deviation. The statistical analysis was performed with Graph-Pad Prism 5.0 (GraphPad Prism 5.0 Software Inc., USA) using the Student's *t*-test. *P* < 0.05 was considered to represent a significant difference in all tests.

## 3. Results

All patients (40) included in the trial had Breast Cancer, Invasive Ductal Carcinoma—Stages II A or II B, according to the American Joint Committee on Cancer (AJCC) and the American Cancer Society (ACS) staging systems [2].

The general characteristics of patients and controls who participated in the study are described in Table 1.

**Figure 1:**
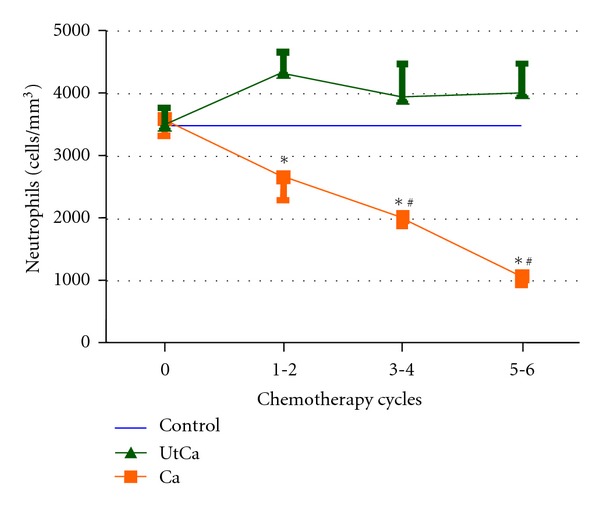
Values neutrophil granulocytes in patients with breast cancer undergoing chemotherapy with (UtCa) and without (Ca) supplementation with *Uncaria tomentosa* and reference values (control). Data are expressed as mean ± standard deviation.

To evaluate the effectiveness of Ut as adjuvant treatment for breast cancer, haematological parameters were used and analyzed (Table 2). At day zero, the results of the haematological parameters analyzed in the blood count did not significantly differ among the Control, the Ca, and the UtCa groups. A greater reduction in the white blood cell (WBCs) and the neutrophil counts were observed in the Ca group along the treatment, differently from the UtCa group, which remained closely the reference values, obtained in the control group (Figure 1). Considering the lymphocytes number, a significant difference between the control group and the groups of patients with breast cancer, either treated or not with Ut in the chemotherapy cycles, was observed. (*P* < 0.05). Monocytes number in patients with breast cancer (treated and not treated with Ut) at 5-6 chemotherapy cycles were higher than control group, but in the UtCa group, this increase was more strong (Table 2).

To evaluate the immune response of patients with breast cancer, CD4^+^ T cells, CD8^+^ T cells (absolute count and ratio) and IL-6 levels were analyzed. During the chemotherapy treatment cycles, no significant difference was observed between groups. There was no difference between groups for any of the parameters analyzed (Table 3).

No correlation between the IL-6, CD4^+^ T/CD8^+^ T ratio and age, body mass index, and hormone receptor status was found (data not shown).

Antioxidant defenses were analyzed by the activity of Superoxide Dismutase (SOD) and Catalase (CAT) compared to treatment cycles zero and six, as well as between the UtCa and the Ca groups. There were no statistically significant differences among groups. An increase in SOD enzyme when compared to treatment cycles zero and six for the group supplemented with Ut was observed, but that difference was not observed between the groups (UtCa = 11.53 U/mg protein, Ca = 11.43 U/mg protein) or at the end of treatment (17.32 U/mg protein, 11.74 U/mg protein). Lipid Peroxidation was also estimated by the TBARS scale and the carbonylation of serum proteins, but there was no difference between groups (UtCa and Ca).

**Figure 2:**
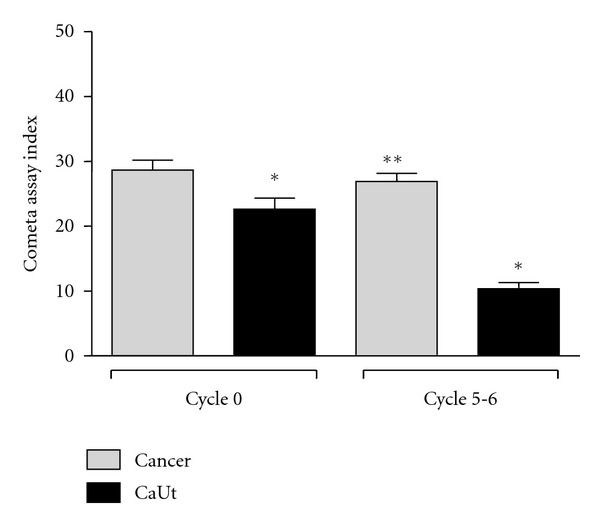
Index test of blood cells in patients with breast cancer treated and not treated with Ut. *Represents significant difference between all groups (*P* < 0.05). **Represents significant difference between the UtCa group (cycle 0) and the UtCa group (cycle 5-6) *P* < 0.05 (Student's *t*-test).

The protective effect of chemotherapy to extract Ut was evaluated by the Comet Assay. In the start of the treatment (zero cycle), the Ca group and UtCa group showed no significant difference in the Comet assay index. However, in the sixth cycle (end of the treatment), it was observed a significant decrease in the index test in the UtCa group, when compared to the Ca group (*P* < 0.05) Figure 2.

## 4. Discussion

*Uncaria tomentosa* enables the stimulation of the immune system, increasing resistance to diseases when the body is immunosuppressed due to stress, malnutrition, or due to the effect of some medication.

Many herbal medicines are used for various purposes, in various combinations (along with allopathic and homeopathic, medicines, etc.) based on historical or personal evidences generally not being associated with any adverse effects [26]. Therefore, this study, through a randomized clinical trial, evaluated the efficacy of Ut as a complementary therapy to chemotherapy.

The cytotoxic effect of chemotherapeutic agents is not selective for neoplastic cells, being also harmful to other body cells. Hematopoietic suppression is the major complication limiting dosage of such cytostatic agents; neutropenia and thrombocytopenia are the most frequent ones [1]. Treatment should be discontinued when neutrophil count is below 500 cells/mm^3^ [27]. Thus, the success of the treatment process depends on the neutrophils content. Prevention of chemotherapy-induced neutropenia should be considered a clinical priority [28]. Once it is known that neutropenia predisposes to serious infections, often resulting in delays in treatment cycles and dose reductions.

Treatment using a daily dose of 300 mg dry Ut extract was effective in reducing the main chemotherapy effect, which is neutropenia. The effects of chemotherapy on blood cells tend to become more pronounced during treatment. However, our results show that in cycle six, which corresponds to the end of chemotherapy, the differences in the leukocytes and neutrophils counts were even more significant, as the group that was supplemented with Ut presented values twice as high of neutrophils when compared to the cancer group (without supplementation). In the group without supplementation, 67.89% of patients had neutropenia. Similarly, there was an increase in activated monocytes, as the activated precursors were common to both strains.

Our findings are corroborated by other studies that had already shown that the Ut extract has a stimulating effect on growth and differentiates the CFU-GM from mice bone marrow and spleen, using the model for listeriosis [29]. Increased leukocytes numbers were also detected using Ut aqueous extract for six consecutive weeks in volunteers [13]. The recovery of leukocytes was also observed in mice using a model for chemotherapy-induced leukopenia (Doxorubicin) using Granulocyte Colony-Stimulating Factor (Neupogen) as a positive control [15].

 Our group confirmed these results using a model for ifosfamide-induced neutropenia in mice, which caused a severe neutropenia. Bioassays showed that treatment with Ut significantly increased neutrophils counts, and a power of 85.2% was calculated in relation to Filgrastim (rhG-CSF) at the corresponding doses tested (5 and 15 mg/day of Ut, and 3 and 9 mcg/day Filgrastim, resp.) [13]. Through *in vitro* assays in human hematopoietic stem precursor cells (hHSPCs) obtained from umbilical cord blood (UCB), we reach the conclusion that this effect happened due to proliferation of Forming Units-Granulocyte-Macrophage (CFU-GM) [13].

In this study, no differences were observed in lymphocyte counts between groups, either supplemented or not with Ut, over the chemotherapy cycles; however, its counts presented decrease due to chemotherapy when compared to the control group. These differences were not observed in the CD4+ and CD8+ subpopulations.

This paper reports the effects of different *Uncaria tomentosa* extracts. The aqueous extract that has the highest concentration of quinic acid and low concentrations of oxindole alkaloids, being related to immunomodulatory properties is mediated by cytokines such as TNF-*α* [30]. Clinical studies using 20 mg/day of *Uncaria tomentosa* extract for 2 to 5 months in patients with HIV, receiving no other therapy, showed an increase in total peripheral lymphocytes without significant changes in the proportion of CD4+ and CD8+ [31]. Healthy volunteers receiving 350 mg of aqueous Ut extract for 8 weeks showed leukocytosis, with a tendency to higher proliferation of lymphocytes [15]. In an animal model, using aqueous extract, which has the highest concentration of quinic acid and low concentrations of oxindole alkaloids, an increase in lymphocytes was also observed [15, 32].

Furthermore, alcoholic extracts and/or pentacyclic oxindole alkaloids have higher myeloproliferative effects [33, 34]. Other studies have shown that the increase in the lymphocyte counts happens due to increased survival rates rather than proliferation [32].

 Thus, changes observed in lymphocytes are associated with the chemically active components defined as quinic and bioactive acid esters *in vivo*, as quinic acid present in the aqueous extract used by authors. In our study, hydroalcoholic extract was used.

High levels of circulating IL-6 are associated with worse survival rates for patients with metastatic breast cancer, being correlated to the extent of the disease [30].

The patients who comprised our sample did not present a negative progression during the treatment cycles, that is, there was no occurrence of relapses or increased lesion extent, which could lead to an increase in IL-6.

Different *Uncaria tomentosa* extracts were tested *in vitro* in order to determine their antioxidant activity. Aqueous and alcoholic extracts prevent the production of reaction products with thiobarbituric acid (TBARS) and, therefore, damage the cytoplasmic membrane (lipids) and DNA by the nonformation of free radicals [35, 36] among the evaluated parameters of oxidative stress, such as SOD, CAT, TBARS, and carbonylated proteins.

Women with breast cancer present an increase in blood concentrations of oxidized substances, such as products derived from lipids peroxidation, proteins, and DNA [32, 33].

The only observed differences were in the SOD enzyme between groups, either with or without supplementation with Ut. These results were also found in an animal model, where an increase in the activity of this enzyme [10] was perceived. In a study on women with breast cancer, SOD activity showed a significant increase regardless of clinical stage and menopausal status [31]. There is evidence that the state of oxidative stress is higher than the greater degree of the disease stage is [37, 38].

Similar to results found in IL-6, the fact that all patients in the study had Stage II cancer may explain the results found.

 The ability of doxorubicin to bind itself to the cell membrane lipid can affect a variety of cellular functions. The reaction of the doxorubicin enzymatic reduction by a variety of oxidase, reductase, and dehydrogenases genes generates ROS and, thus, may result in damage to DNA and proteins, triggering apoptosis [39, 40].

The performance of antioxidants *in vivo* depends on the types of free radicals formed, where and how these radicals are generated, and what are the doses for optimal protection. So it is entirely possible for an antioxidant to act as a protector in any given systems, but it is also possible for it to fail to protect, or even increase lesions induced in other systems or tissues. Thus, the use of antioxidants in cancer treatment is controversial.

Ambrosone and colleagues [41] observed that women having breast cancer with genotypes that result in higher levels of ROS had better survival rates than those with genotypes associated with lower generations of ROS. Such results indicate that an increased oxidative stress may increase the effects of chemotherapy and/or radiotherapy, resulting in improved treatment efficacy and, thus, better survival rates. The overexpression of SOD is associated with better survival rates for patients diagnosed with colorectal cancer [42].

 Cleveland and Kastan suggest that a promising treatment for some types of cancer could happen by increasing ROS levels and inhibiting SOD levels [43, 44]. Other authors report that the overexpression of SOD has presented resistance to doxorubicin [43, 44], but not 5-fluorouracil in gastric cells [41]. Another study on breast cancer cells showed an increased resistance to Adriamycin with the intracellular level of glutathione (GSH) [45].

 The protective effect of Ut on DNA was observed during the breast cancer cycles of treatment, by the Comet test analysis.

Doxorubicin has its own mechanism of action related to its binding to the DNA and the inhibition of nucleic acid synthesis. Studies have shown that aqueous Ut extracts present DNA repairing activities [15]. Mammone et al. (2006) showed the ability to modulate *Uncaria tomentosa* and repair of DNA in human skin and organ cultures [46].

In the present study, the results of comet test suggest that Ut had a protective effect of the DNA during the treatment cycles. However, it is necessary for other studies to confirm these effects.

## 5. Conclusions

*Uncaria tomentosa*, used at dose of 300 mg dry extract per day, is effective in the recovery from neutropenia induced by chemotherapy in women diagnosed with Invasive Ductal Carcinoma—Stage II. It is also able to restore cellular DNA. Thus, it is a safe and effective adjuvant treatment in reducing adverse chemotherapy effects.

## Figures and Tables

**Table 1:** Clinical characteristic of patients. It represents age, body mass index (BMI), total cholesterol levels, estrogen receptor (ER), and progesterone receptors (PR), as well as the HER-2 status in different groups.

Clinical parameters	Control (*n* = 20)	Ca (*n* = 20)	UtCa (*n* = 20)
Age interval	32–79	32–71	40–75
Mean age	56.5 ± 11.6	55.0 ± 9.7	54.4 ± 11.0
BMI	25.0 ± 1.93	27.27 ± 1.49	26.82 ± 5.03
Cholesterol levels	202.5 ± 1.90	238.9 ± 57.9	244.2 ± 44.5
Estrogen receptor status (ER)			
Positive	—	+14	+17
Negative	—	−6	−3
Progesterone receptor status (PR)			
Positive	—	+10	+11
Negative	—	−10	−9
HER-2 receptor status (HER2)^∗^			
Positive	—	+2	+6
Negative	—	−16	−12

The results for ER, HER2, and REP are represented as positive and negative numbers for the expression of receptors by number of women, while other parameters are expressed as mean ± standard deviation. UtCa group: patients treated with chemotherapy +300 mg *Uncaria tomentosa* daily (*n* = 20); Ca group: patients received chemotherapy (*n* = 20); control group (*n* = 20).

^∗^To HER-2 receiver only data were obtained from 18 patients.

**Table 2:** Leukocytes, neutrophils, lymphocytes, and monocytes levels in breast cancer patients before treatment and after 6 cycles of chemotherapy without *Uncaria tomentosa* supply (Ca group) or receiving 300 mg/day of *Uncaria tomentosa* (UtCa group).

Parameters	Cycles			
(cells/mm^3^)	0	1-2	3-4	5-6
Leukocytes				
Control	6800 ± 1458			
UtCa	6800 ± 1458	7890 ± 1615	6636 ± 2578	5469 ± 1626
Ca	6653 ± 1158	6617 ± 1504	4092 ± 1047^∗#^	3247 ± 1117^∗#^
Neutrophils				
Control	3510 ± 1077			
UtCa	3496 ± 1108	4335 ± 1626	3937 ± 1992	4016 ± 1545
Ca	3588 ± 1081	2663 ± 1351*	2028 ± 512^∗#^	1083 ± 368^∗#^
Lymphocytes				
Control	2264 ± 490,6			
UtCa	2276 ± 503,3	2376 ± 708,1	1627 ± 578,7^∗^	1411 ± 596,6^∗^
Ca	2177 ± 453,3	2284 ± 867,9	1460 ± 512,5^∗^	1208 ± 395,1^∗^
Monocytes				
Control	487,6 ± 128,9			
UtCa	515,3 ± 169	560 ± 322	814,9 ± 309^#^	817 ± 444,6
Ca	541,6 ± 161	526,6 ± 154	654,1 ± 310^∗#^	500,9 ± 226^∗#^

Data expressed asmean ± standard deviation.

^∗^Represent difference significant between the Ca and UtCa groups, *P* ****<** **0.05*.*

^#^Represent difference significant of the control group, *P* ****<** **0.05 (Student's *t*-test).

**Table 3:** Immune status of breast cancer patients before treatment and after 6 cycles of chemotherapy without *Uncaria tomentosa* supply (Ca group) or receiving 300 mg/day of *Uncaria tomentosa* (UtCa group).

Parameters	Group	Chemotherapy cycles	
		0	6
CD4^+^ T cells	UtCa	1008.25 (379.21)	786.60 (310.49)
Cells/*μ*L	Ca	1053.00 (620.81)	798.00 (366.14)

CD8^+^ T cells	Ut Ca	568.81 (295.60)	459.87 (246.46)
Cells/*μ*L	Ca	679.84 (273.22)	565.62 (231.05)

CD4^+ ^T/CD8^+ ^T ratio	UtCa	2.044 (0.62)	1.858 (0.89)
	Ca	1.630 (0.69)	1.652 (0.49)

IL6	UtCa	3.4 (4.50)	2.1 (6.6)
pg/mL	Ca	5.6 (5.53)	3.8 (7.312)

Data expressed as mean ± standard deviation; UtCa group: patients treated with chemotherapy +300 mg *Uncaria tomentosa* daily (*n* = 20); Ca group: patients received chemotherapy (*n* = 20).
